# Effects of Dipsacus asperoides Extract on Monosodium Iodoacetate–Induced Osteoarthritis in Rats Based on Gene Expression Profiling

**DOI:** 10.3389/fphar.2021.615157

**Published:** 2021-04-13

**Authors:** Jin Mi Chun, A Yeong Lee, Jae Yong Nam, Kyung Seob Lim, Mu Seog Choe, Min Young Lee, Chul Kim, Joong-Sun Kim

**Affiliations:** ^1^ Herbal Medicine Resources Research Center, Korea Institute of Oriental Medicine, Naju-si, Korea; ^2^ Bioinformatics Group, R&D Center, Insilicogen Corporation, Yongin, Korea; ^3^ Futuristic Animal Resource and Research Center, Korea Research Institute of Bioscience and Biotechnology, Cheongju-si, Korea; ^4^ Department of Molecular Physiology, College of Pharmacy, Kyungpook National University, Daegu, Korea; ^5^ Korea Future Medicine Division, Korea Institute of Oriental Medicine, Daejeon, Korea

**Keywords:** *Dipsacus asperoides* ethanolic extract, Traditional herbal medicine, Osteoarthritis, Monosodium iodoacetate, Transcriptomic analysis

## Abstract

The root of *Dipsacus asperoides* C. Y. Cheng et T. M. Ai is traditionally used as an analgesic and anti-inflammatory agent to treat pain, rheumatoid arthritis, and bone fractures. However, neither its effects on osteoarthritis (OA) nor its effects on the arthritic cartilage tissue transcriptome have not been fully investigated. In this study, we used a rat model of monosodium iodoacetate- (MIA-) induced OA to investigate the therapeutic effects of a *Dipsacus asperoides* ethanolic extract (DAE, 200 mg/kg for 21 days). The study first assessed joint diameter, micro-CT scans, and histopathological analysis and then conducted gene expression profiling using RNA sequencing in articular cartilage tissue. We found that DAE treatment ameliorates OA disease phenotypes; it reduced the knee joint diameter and prevented changes in the structural and histological features of the joint, thereby showing that DAE has a protective effect against OA. Based on the results of gene expression profiling and subsequent pathway analysis, we found that several canonical pathways were linked to DAE treatment, including WNT/β-catenin signaling. Taken together, the present results suggest molecular mechanism, involving gene expression changes, by which DAE has a protective effect in a rat model of MIA-induced OA.

## Introduction

The root of *Dipsacus asperoides* C. Y. Cheng et T. M. Ai (family Caprifoliaceae) is a traditional medicine listed in the Korean Pharmacopoeia and Chinese Pharmacopoeia (KIOM, 2020). According to the Korean Medicine Classification of Efficacy, *Dipsacus asperoides* is a tonifying and replenishing medicine or a yang-tonifying medicine that is traditionally used as an analgesic and anti-inflammatory agent to treat pain, rheumatoid arthritis, and bone fractures (Gong et al., 2019). It is also traditionally used in Chinese Medicine for the treatment of bone fractures and other bone diseases (Wong et al., 2007; He et al., 2019). Recent studies show that *Dipsacus asperoides* has several beneficial actions, such as antioxidant and anti-inflammatory effects (Park et al., 2019), as well as antiasthmatic effects (Shin et al., 2019). Several reports show that *Dipsacus asperoides* has a substantial antirheumatic effect on collagen-induced arthritis in mice (Jung et al., 2012) and an osteoprotective effect in ovariectomized rats (He et al., 2019; Sun et al., 2019). In addition, Chinese herbal medicine paste containing *Dipsacus asperoides* relieves osteoarthritic knee pain in rats (Siu et al., 2019); these effects are related to the ethnopharmacological actions of the plant. However, the ethnomedicinal effects of *Dipsacus asperoides* on the joint disease osteoarthritis (OA) have not yet been reported, even though natural product research for OA is an area of active research.

OA is the most common type of degenerative joint disease. It is caused by destruction of joint cartilage and underlying bone and is a disease that affects the entire joint. The prevalence and risk of OA increase rapidly with age (Martel-Pelletier et al., 2016; Chen et al., 2017; Hunter and Bierma-Zeinstra, 2019). OA is characterized by multiple risk factors and is a complex disease involving cellular and molecular mechanisms, metabolic processes, and inflammation that together are responsible for disease occurrence and progression (Lee et al., 2013; Sokolove and Lepus, 2013). Therefore, OA is difficult to treat, and research on definition, risk factors, causes, and pathophysiology is ongoing. In addition, many natural products and herbal medicines exhibit pharmacological actions suitable for the treatment of experimental OA models (Mével et al., 2016; Chun et al., 2018).

Although current treatments for OA are limited, various attempts to develop new treatment strategies for OA based on functional genomic analysis are underway (Thysen et al., 2015). Recent studies using functional genomic analysis of the mechanisms of herbal medicines have increased our understanding of the multitarget mechanisms involved in OA (Liu et al., 2016b; Baek et al., 2018; Seo et al., 2018; Wang et al., 2018). Since natural products can have multitarget and multicomponent effects, it is necessary to use transcriptomic analysis, to fully understand the overall molecular mechanisms that underlie any beneficial effects of *Dipsacus asperoides* on OA.

In this present study, we investigated the effects of a DAE on OA and its possible mechanism of action using gene expression profiling. First, the effects of DAE on joint edema, joint morphology, and histopathological features were evaluated in rats with a monosodium iodoacetate- (MIA-) induced OA. Then, the mechanisms underlying the effects were examined using gene expression profiling of articular cartilage tissue to investigate the involvement of regulatory genes and signaling pathways.

## Materials and Methods

### Plant Materials and Chemicals

The root of *Dipsacus asperoides*, which was purchased from NaemomeDah Herbal Medicine (Ulsan, Korea), was authenticated using morphological feature analysis (Dr. Goya Choi, Korea Institute of Oriental Medicine [KIOM]) and genetically identified by sequence characterized amplified region (SCAR) marker analysis as described previously (Park et al., 2018). The voucher specimen was deposited in the Korean Herbarium of Standard Herbal Resources (No. 2-17-0059 and 2-17-0060).

Six DAE standards were used and obtained from the following suppliers: chlorogenic acid (≥98%) and loganin (≥98%) were purchased from KOC Biotech Corporation (Daejeon, Korea) and Wako Pure Chemical Corporation (Osaka, Japan), respectively; loganic acid (≥98%), sweroside (≥97%), and isochlorogenic acid A (≥98%) were obtained from ChemFaces Biochemical Co., Ltd. (Wuhan, Hubei, China); and akebia saponin D (≥95%) was obtained from National Development Institute of Korean Medicine (Gyeongsangbuk-do, Korea). HPLC grades of acetonitrile, methanol, and distilled water were purchased from Merck (Darmstadt, Germany).

### Preparation of *Dipsacus asperoides* Ethanolic Extract

DAE (608.9 g) was pulverized using a blender (SHMF-3000S, Hanil Electric, Seoul, Korea) and refluxed in 70% ethanol for 2 h. The extract was filtered through chromatography paper (46 × 57 cm, Whatman Ltd., Maldstone, England). After removal of the ethanol *in vacuo*, the residue was dried in a freeze-dryer at −78°C, as described previously (Shin et al., 2019). The yield of DAE was 47.9% (*w/w*), and it was stored at −20°C until use. The lyophilized powder was dissolved in 0.25% carboxymethyl cellulose before use.

### HPLC Analysis of *Dipsacus asperoides* Ethanolic Extract

To quantify the components of DAE, DAE (85.0 mg) was dissolved in 70% ethanol (10 ml) and filtered through a syringe filter, and reference compounds were prepared as stock solutions. The stock solutions were dissolved in 70% ethanol (10 ml). Serial dilutions were performed to construct standard calibration curves with compound concentrations of 0.4–250 or 0.8–500 or 91.875–1470 μg/ml. HPLC analysis was performed on a chromatographic system equipped with a separation module (e2695) and a 2998 photometric diode array detector followed by a quadrupole detector (Waters Co., Milford, MA, United States). Analytical data were processed using the Empower 3.0 program (Waters Co., Milford, MA, United States). Liquid chromatography separations were performed on a Kinetex phenyl-hexyl column (5 μm, 4.6 × 250 mm, Phenomenex Inc., Torrance, CA, United States) with 10 μl injected volume and 0.8 ml/min flow rate. The mobile phase consisted of 0.05% formic acid in distilled water, methanol, and acetonitrile, and the linear gradient program was from 90% water (1% methanol and 9% acetonitrile) to 0% water (8% methanol and 92% acetonitrile) for 40 min. Single quadruple mass detector (QDa detector) conditions were set as follows: probe temperature 600°C, ESI capillary voltage 0.8 kV, con voltage 15 V, source temperature 121°C, and split ratio 20:1. The sampler and column temperatures were set at 4°C and 30°C, respectively. The wavelength was scanned from 195 to 400 nm, and sample peaks were detected at 200, 240, and 320 nm. DAE peaks from HPLC analysis were compared with standard peaks in terms of retention time, ultraviolet wavelength, and mass spectra.

### Method Validation for LC-MS

The linearities of chlorogenic acid, loganin, loganic acid, sweroside, isochlorogenic acid A, and akebia saponin D were evaluated using a correlation coefficient-derived calibration curve. The limit of detection (LOD) and limit of quantification (LOQ) were determined using the signal-to-noise approach in Empower 3.0 software (Waters Co., Milford, MA, United States). In general, signal-to-noise ratios of LOD and LOQ were 3:1 or 2:1 and 10:1, respectively.

### Animals and Experimental Procedures

Male Sprague-Dawley rats (7 weeks old) were purchased from Daehan Bio Link, Inc. (Eumseong, Chungcheongbuk-do, Korea), and maintained at an air-conditioned temperature on a 12 h light–dark cycle with free access to food and water. After acclimatization for 1 week, rats were randomly assigned to one of three experimental groups (*n* = 8 per group) as follows: 1) saline only (vehicle), 2) MIA-treated rats (the MIA model), 3) MIA + DAE-treated rats (rats treated with both MIA and DAE). Animal experiments were approved by the Ethics Committee of Kyungpook National University (KNU 2018-0091).

### Induction of Osteoarthritis and Measurement of Knee Diameter

The MIA-induced rat model of OA was performed as previously described (Combe et al., 2004). To induce OA, the knee joints of rats were injected with MIA (60 mg/ml saline) via intra-articular injection (Schuelert and McDougall, 2009). Immediately after MIA induction, vehicle or DAE (200 mg/kg) was orally administered daily to rats for 21 days. A DAE dose of 200 mg/kg was selected as the effective dose, based on previous reports of DAE being nontoxic and effective dosage (Li et al., 2019)^1^.

To monitor OA symptoms, the joint diameter of rats’ knees was accessed with a digital caliper (Mitsutoyo Co., Ltd., Tokyo, Japan) weekly for 21 days. The change in (delta) mean knee diameter was calculated as described previously (Fernihough et al., 2004; Ishikawa et al., 2015). At the end of the experiment, all rats were sacrificed, and representative knee joint tissues were fixed in 10% formalin for micro-CT scanning and/or histological analysis. Articular cartilage was rapidly isolated from other joint tissues and stored at −70°C for use in molecular assays.

### Micro-Computed Tomography and Histopathological Analysis

For the evaluation of structural characteristics, rat knee joints were analyzed using a Biograph mCT Series PET/CT Scanner imaging system (Siemens, United States) of the subchondral bone architecture of rats prior to histopathological analysis.

Following micro-CT scanning, tissues were fixed, decalcified, dehydrated, embedded in paraffin, and serially sectioned for histological analysis. To observe changes in joint cells, matrices and proteoglycans in articular cartilage tissue sections were stained with hematoxylin and eosin (H&E) and safranin O. Histological changes were observed by light microscopy using an Olympus CX31/BX51 instrument (Olympus Optical Co., Tokyo, Japan) and an Olympus DP70 camera (Olympus). Quantitative analysis of histological assessment was evaluated by the Osteoarthritis Research Society International (OARSI) scoring system as described previously (Udo et al., 2016).

### Immunohistochemical Analysis

Immunohistochemical analysis was performed on paraffinized and rehydrated sections with specific primary antibodies. Unstained sections were incubated with rabbit monoclonal anti-matrix metalloproteinase- (MMP-) 9 (1:100; #13667s, Cell Signaling Technology, MA, United States) for 2 h at room temperature (RT). Sections were then incubated with biotinylated goat anti-rabbit IgG for 1 h at RT, and immunoreactivity was detected after 1 h at RT using an avidin-biotin peroxidase complex and a VECTASTAIN ABC Elite kit (Vectorlabs, CA, United States) according to manufacturer’s instructions. The peroxidase reaction was developed using an SK-4100 diaminobenzidine substrate kit (Vectorlabs) followed by light microscopy with an Olympus CX31/BX51 instrument (Olympus Optical Co) and photography using an Olympus DP70 camera (Olympus).

### RNA Extraction, Library Preparation, and RNA Sequencing

Total RNA was isolated from articular cartilage tissue using a mirVana™ miRNA isolation kit (Life Technologies, Grand Island, NY, United States). Total RNA from purified samples was treated with DNase, and RNA purity was measured using a NanoDrop 8000 spectrophotometer (Thermo Fisher Scientific, Wilmington, DE, United States). For RNA-seq, the quality and quantity of the RNA samples, including the assignment of a RNA quality indicator number, were determined with an Agilent Technologies 2100 Bioanalyzer (Agilent Technologies, Santa Clara, CA, United States), and samples with an RNA Integrity Number (RIN) >7.0 were used. Total RNA sequencing (RNA-seq) libraries were constructed using the Truseq stranded total RNA sample preparation kit (Illumina, San Diego, CA, United States) according to the manufacturer’s protocol. Library pools and clusters were generated on a cBot clonal amplification system (Illumina) and sequenced on the Novaseq 6000 sequencing system (Illumina) with 2 × 100 bp paired-end reads.

### Analysis of RNA-Seq Data

Reads were trimmed to remove adapters and low-quality reads, and high-quality reads were aligned to the *Rattus norvegicus* reference genome (RGSC 6.0/rn6) by Hisat2 (v. 2.1.0). StringTie (v. 1.3.4) (Pertea et al., 2015; Pertea et al., 2016) was then used to correctly assemble transcripts, and differentially expressed genes (DEGs) among the experimental groups were analyzed by the ballgown R package (v. 2.12.0) (Frazee et al., 2014). DEGs were identified using a cutoff threshold *p-value* of <0.05 and log2 fold change >2 and were then subjected to Ingenuity Pathway Analysis.

### Ingenuity Pathway Analysis

RNA-seq data were analyzed using Ingenuity Pathway Analysis (1.13; Qiagen, Valencia, CA, United States) software for identifying the function and pathways associated with DEGs. Each DEG associated with MIA and DAE treatment was mapped to a corresponding gene object in the Ingenuity Pathway Analysis knowledge base, and analysis was performed on transcriptomic datasets to generate canonical pathways and to identify the biological functions, networks, and pathways of the genes affected by DAE treatment.

### Real-Time Quantitative Polymerases Chain Reaction Analysis

Quantitative polymerases chain reaction (qPCR) analysis was performed to validate the mRNA expression levels of selected DEGs identified from RNA-seq data. RNA extraction was carried out as described in the RNA Extraction, Library Preparation, and RNA Sequencing section. Template RNA was reverse-transcribed to synthesize cDNA using a ReverTra Ace qPCR RT kit (Toyobo, Tokyo, Japan). The PCR reaction (20 μl) system used a SsoAdvanced universal SYBR Green supermix (Bio-Rad, Hercules, CA, United States) with 4 μl cDNA (80 ng), 10 μl SYBR mixture, 1 μl of each of the forward and reverse primers (10 μM), and 4 μl deionized water. After mixing, the PCR reaction was performed on a CFX96TM real-time system (Bio-Rad, Hercules, CA, United States). The PCR conditions were at 95°C for 5 min, followed by 45 cycles at 94°C for 30 s, 60°C for 30 s, and 72°C for 30 s. The results were normalized using β-actin as a reference gene. This gene is considered to be commonly used in bone and cartilage RT-qPCR analysis and as the cartilage tissue-specific top-ranked reference candidate by referring to previous reports (Yüzbaşioğlu et al., 2010; Cappato et al., 2019; Lv et al., 2019), and relative gene expression data were analyzed using the 2^−△△Ct^ method. All of the samples were analyzed in triplicate. All of the primer sequences for selected genes are listed in Supplementary Table S1.

### Statistical Analysis

The data are presented as the means ± standard deviation (SD). Comparisons between the three rat groups were analyzed by one-way analysis of variance (ANOVA) with Dunnett's multiple comparison test or two-way ANOVA with Tukey’s multiple comparison test. Statistical analyses were performed using GraphPad Prism Software version 7.0 (GraphPad Software, La Jolla, CA, United States). The results were considered statistically significant at a *p*-value <0.05.

## Results

### Phytochemistry Properties of *Dipsacus asperoides* Ethanolic Extract

For definitive authentication and quality control, *Dipsacus asperoides* was authenticated using morphological characterization and SCAR markers, followed by phytochemical analysis. Prior to quantitative analysis, the six components of DAE were validated using an analytical method. The calibration curves showed excellent linearity with correlation coefficients of 1.000 for all compounds, and the LOD and LOQ values of these compounds were confirmed (Table 1).

**TABLE 1:** Method validation parameters of HPLC including linearity, limit of detection (LOD), and limit of quantification (LOQ) of six components.

Analytes	Calibration curves	Correlation coefficients	Compound concentrations (μg/ml)	LOD (μg/ml)	LOQ (μg/ml)
Loganic acid	Y = 11183.16X + 7144.23	1.0000	0.4–250	0.08	0.33
Chlorogenic acid	Y = 36543.53X + 60274.96	0.9998	0.8–500	0.016	0.08
Loganin	Y = 19786.47X + 15348.67	0.9999	0.4–250	0.067	0.2
Sweroside	Y = 14150.85X + 7399.24	1.0000	0.4–250	0.04	0.2
Isochlorogenic acid A	Y = 43110.22X + 7419.40	1.0000	0.4–250	0.01	0.08
Akebia saponin D	Y = 3697.89X + 88237.29	0.9999	91.875–1470	4.50	10.95

**FIGURE 1:**
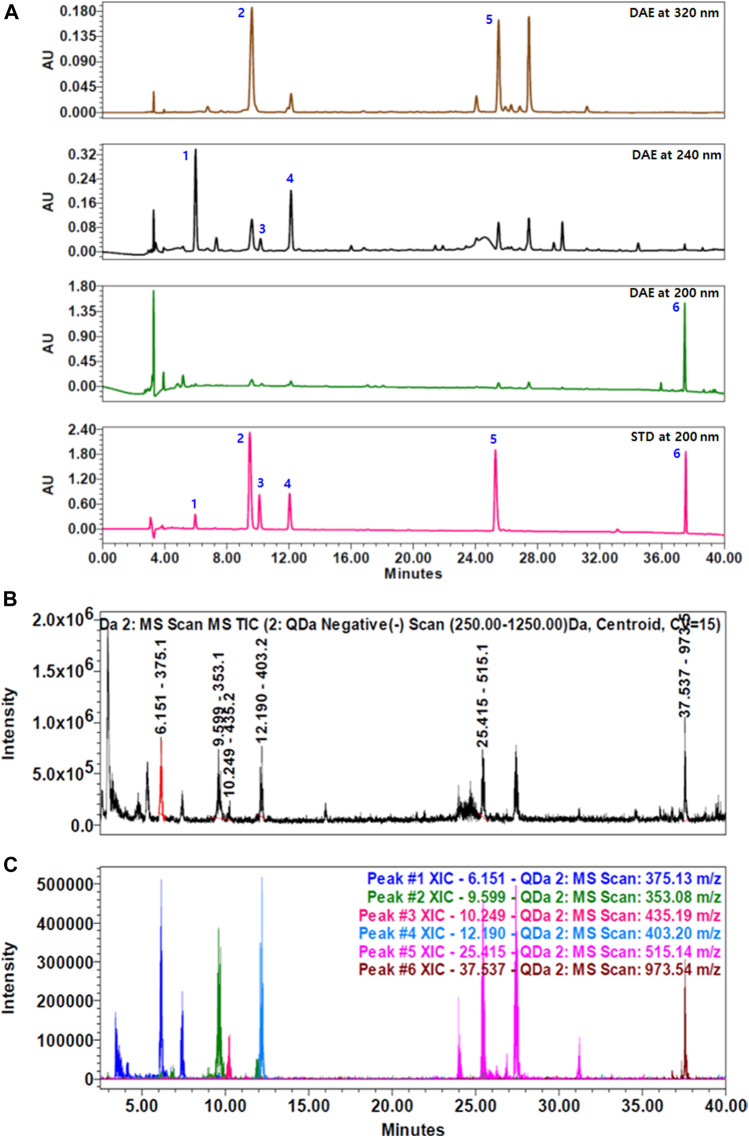
HPLC chromatograms of *Dipsacus asperoides* ethanolic extract (DAE). **(A)** Chromatograms of DAE analyzed by HPLC–PDA at 200, 240, and 320 nm. 1) Loganic acid, 2) chlorogenic acid, 3) loganin, 4) sweroside, 5) isochlorogenic acid, and 6) akebia saponin D. **(B)** Total ion current (TIC) chromatogram and **(C)** extracted ion chromatogram (XIC) of the DAE compounds analyzed by HPLC–MS. MS values (*m/z*) in negative mode of mass spectrum were confirmed as follows: loganic acid [M − H]^-^ = 375.13, chlorogenic acid [M − H]^-^ = 353.08, loganin [M + COOH^−^]^-^ = 435.19, sweroside [M + COOH^−^]^-^ = 403.20, isochlorogenic acid A [M − H]^-^ = 515.14, and akebia saponin D [M + COOH^−^]^-^ = 973.54.

To identify the main components of DAE, we performed quantitative analysis using a HPLC–PDA–QDa system, which detected three iridoids, namely, loganic acid 1), loganin 3), and sweroside 4) at 240 nm, two phenolic acids, namely, chlorogenic acid 2) and isochlorogenic acid A 5) at 320 nm, and one saponin, namely, akebia saponin D 6) at 200 nm (Figure 1A). The concentrations of the compounds were expressed as μg/mg of dry extract. Loganic acid (23.60 ± 0.47 μg/mg) was eluted at 6.01 min, chlorogenic acid (4.61 ± 0.09 μg/mg) at 9.61 min, loganin (2.47 ± 0.07 μg/mg) at 10.18 min, sweroside (9.34 ± 0.54 μg/mg) at 12.11 min, isochlorogenic acid A (3.17 ± 0.01 μg/mg) at 25.33 min, and akebia saponin D (176.90 ± 0.84 μg/mg) at 37.57 min. These DAE components were confirmed by comparing the peaks from the UV spectrum with the peak MS values (*m/z*). Figures 1B,C show the total ion chromatogram (TIC) and extracted ion chromatogram (XIC) of the DAE components, respectively. The main ions in the MS spectrum were 375.13 [M-H]-, 353.08 [M-H]-, 435.19 [M + COOH-]-, 403.20 [M + COOH^−^]^−^, 515.14 [M − H]^−^, 973.54 [M + COOH^−^]^−^ in negative mode and corresponded to loganic acid 1), chlorogenic acid 2), loganin 3), sweroside 4), isochlorogenic acid A 5), and akebia saponin D 6), respectively.

### Protective Effect of *Dipsacus asperoides* Ethanolic Extract on the Knee Joint in Osteoarthritis Rats

**FIGURE 2:**
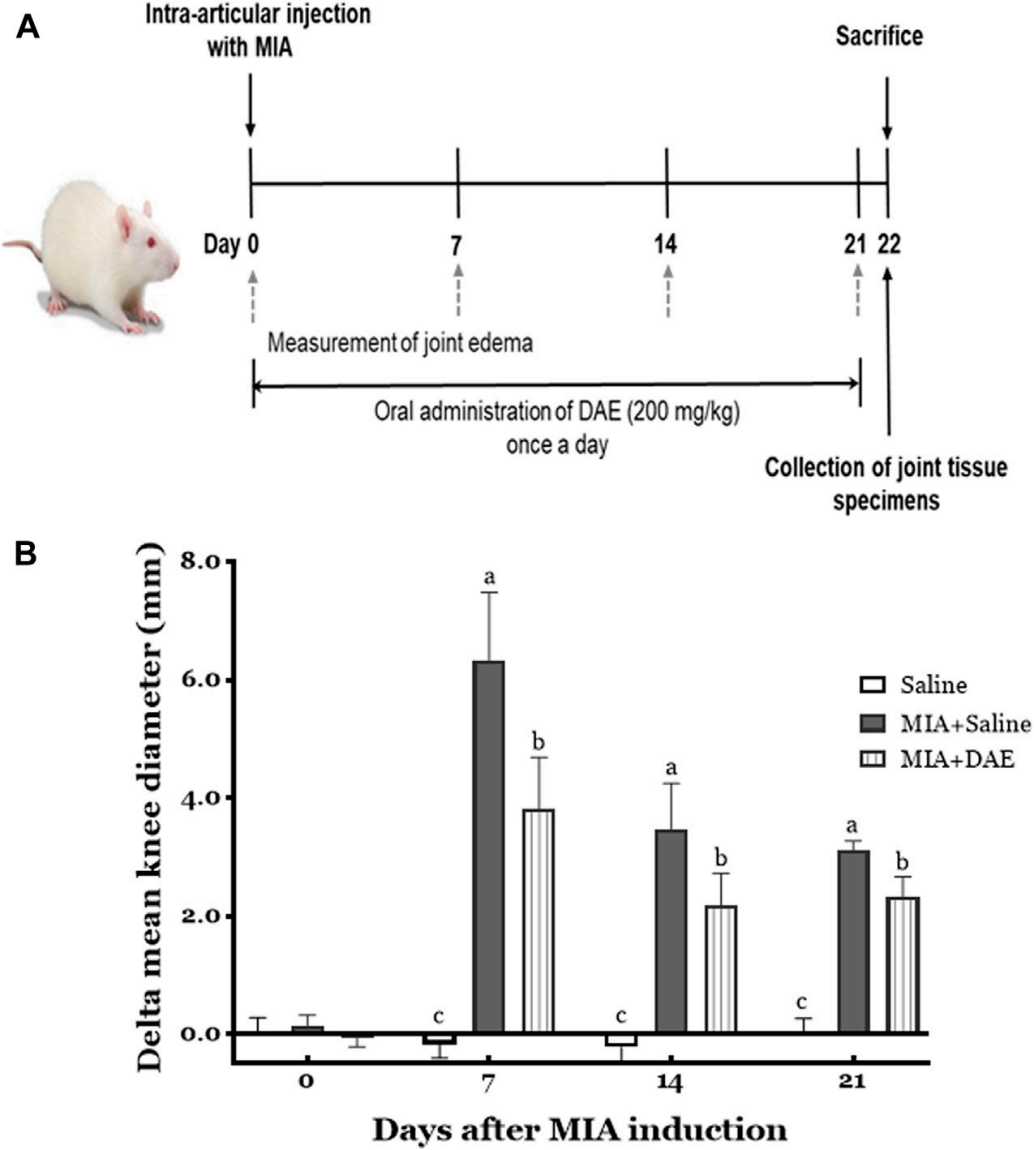
Experimental scheme and effects of *Dipsacus asperoides* ethanolic extract (DAE) on knee diameter in monosodium iodoacetate- (MIA-) induced osteoarthritis in rats. **(A)** The experimental protocol used to induce OA by intra-articular injection with MIA and administration of DAE (200 mg/kg) every day for 21 days. **(B)** After MIA injection, the knee diameter was recorded once per week for 21 days. Index was calculated by subtracting the values of the left side from those of the right. Data are expressed as mean ± SD (*n* = 8 per group). Means (with letters a, b, and c) were significantly different from each other, as determined by two-way analysis of variance (ANOVA) followed by Tukey’s multiple comparison test (*p* < 0.05). Saline, rats given saline (vehicle); MIA + Saline, rats injected with MIA and given saline; MIA + DAE, rats injected with MIA and given DAE.

This experimental scheme of the MIA-induced rat model of OA is summarized in Figure 2A.

The effects of MIA and/or DAE treatment were evaluated by measuring changes in knee diameter as an inflammatory index of joint edema. As shown in Figure 2B, there was no significant change in the diameter of the knee joints in saline-treated rats for 21 days, whereas MIA-treated rats had larger value of difference in mean knee diameters on day 7, which decreased gradually during the experiment; the mean knee diameter in MIA-treated rats was statistically significant compared with the saline-treated group at each time point. MIA and DAE were significantly reduced at all time points compared with MIA-treated rats.

**FIGURE 3:**
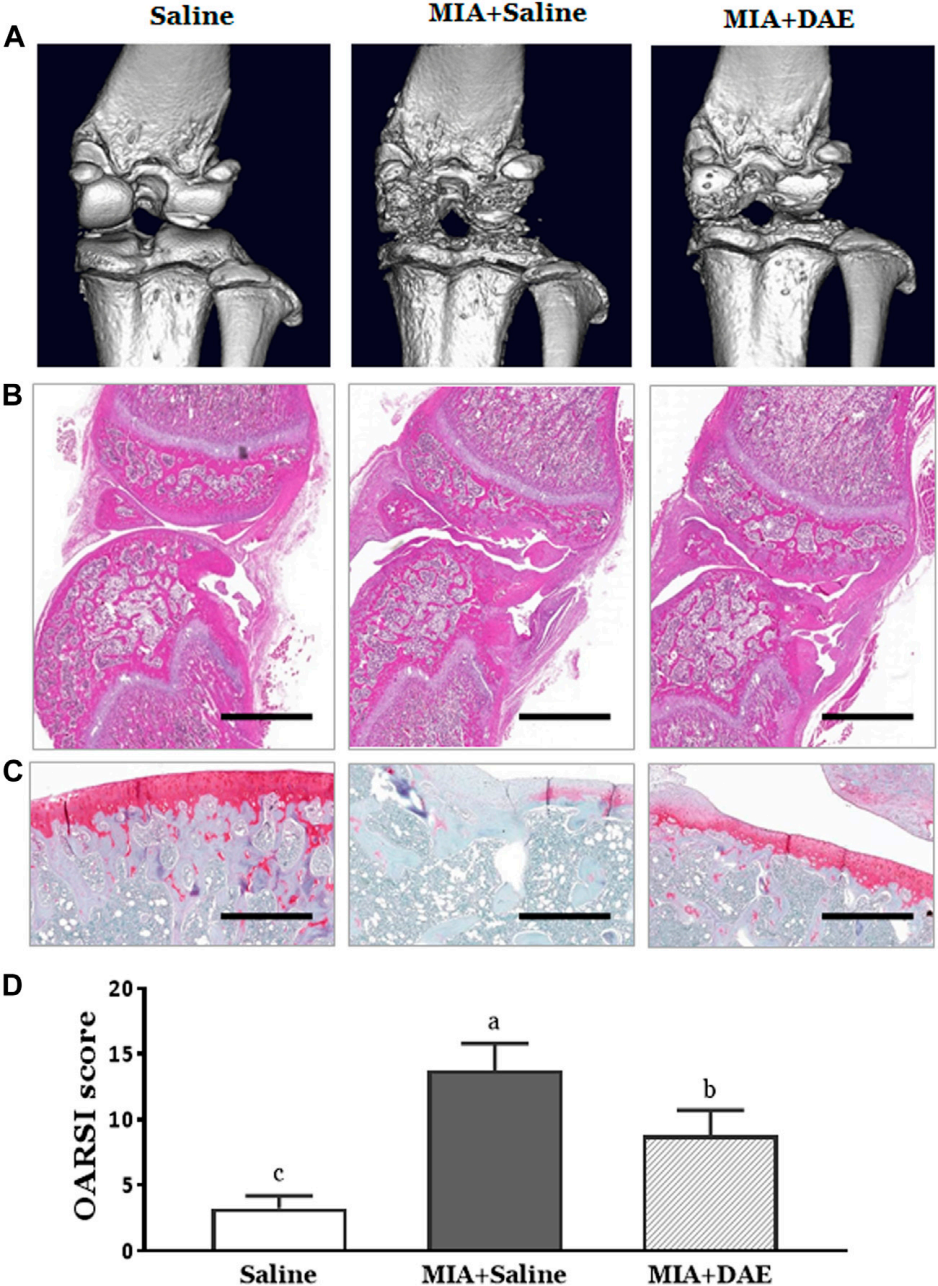
Effects of *Dipsacus asperoides* ethanolic extract (DAE) on micro-computed tomography (micro-CT) images and histopathological features of knee joint lesions in rats with monosodium iodoacetate- (MIA-) induced osteoarthritis. **(A)** Representative *in vivo* micro-computed tomography images of a knee joint. Representative photographs of knee joint sections stained with **(B)** H&E (scale bars = 2,000 μm) and **(C)** safranin O-fast green (×100 magnification, scale bars = 500 μm) and **(D)** OARSI scores of cartilage destruction (*n* = 3 per group). Data are expressed as mean ± SD (*n* = 3 per group). The mean of each group (labeled with letters a, b, and c) was significantly different, as determined by one-way analysis of variance (ANOVA) followed by Dunnett’s multiple comparison test (*p* < 0.05). Saline, rats given saline (vehicle); MIA + Saline, rats injected with MIA and given saline; MIA + DAE, rats injected with MIA and given DAE. OARSI, Osteoarthritis Research Society International.

To observe the effect of DAE on structural and histological changes, micro-CT analysis and histopathological staining were performed on the knee joint region of rats in each treatment group. First, micro-CT image showed that the entire joint region was damaged in MIA-treated rats, including structural damage to the bone and worn cartilage. Rats treated with MIA and DAE tended to have less damage to the cartilage area than the MIA-treated group (Figure 3A). Next, histopathological analysis using H&E and safranin O staining showed severe cartilage changes, such as tissue surface irregularities, matrix loss, articular cartilage damage, and chondrocyte reduction in MIA-treated rats. However, treatment with DAE attenuated these conditions; OARSI score (osteoarthritis cartilage histopathology assessment system), a histopathological grading system of OA cartilage, was lower in MIA/DAE-treated rats (Figures 3B–D). Taken together, these results demonstrate that DAE treatment improved the overall damage to articular cartilage induced by MIA, thereby showing that DAE had protective effects on the knee joint in OA rats.

### Effects of *Dipsacus asperoides* Ethanolic Extract on Gene Expression and Canonical Pathways

**FIGURE 4:**
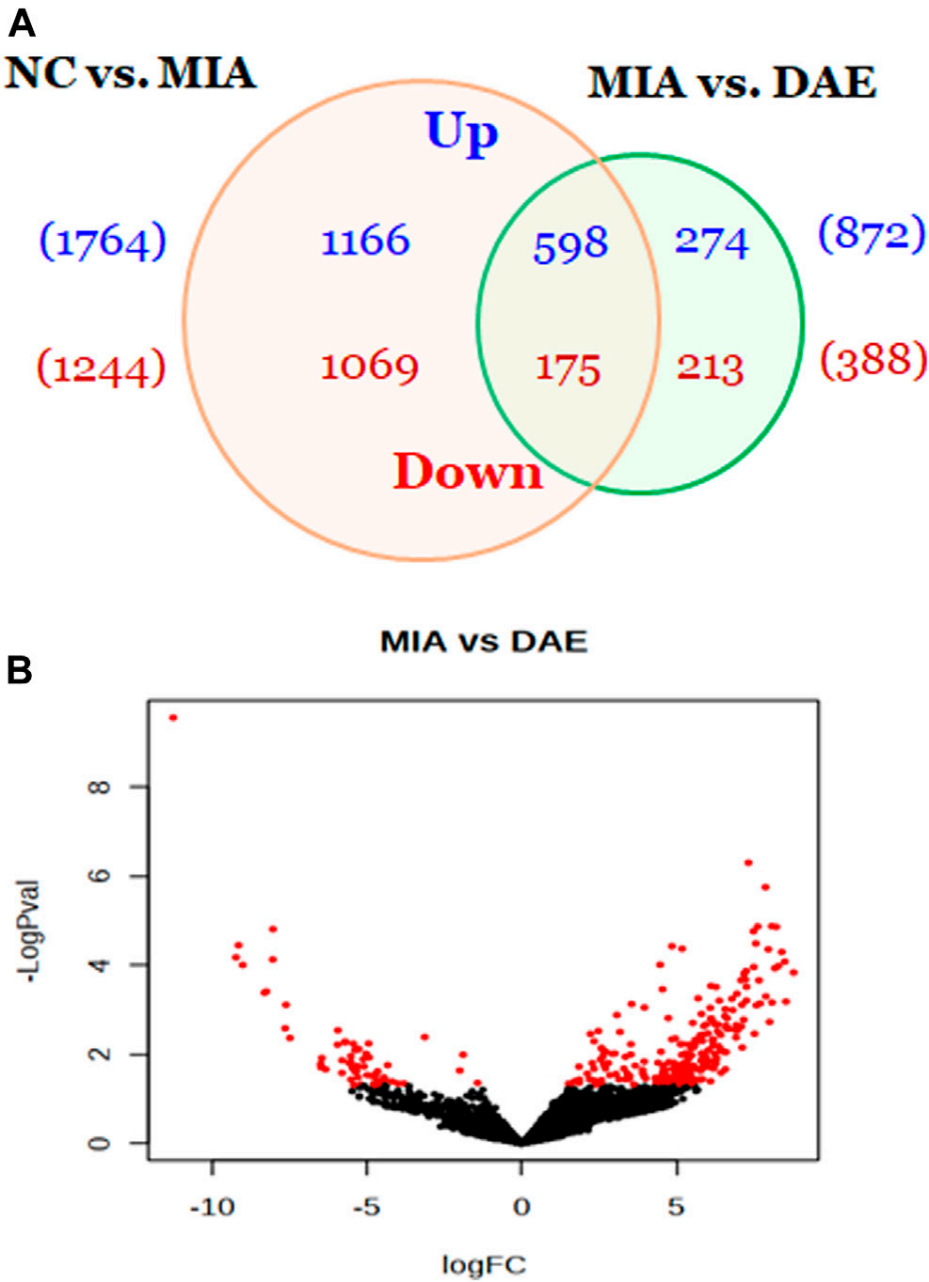
Overview of genes affected by *Dipsacus asperoides* ethanolic extract (DAE) treatment. **(A)** Venn diagram showing the number of up- and downregulated genes in comparisons of saline- vs. MIA-treated rats and MIA- vs. MIA + DAE-treated groups. Genes with fold changes >2 and a *p*-value <0.05 were counted. **(B)** Volcano plot of differentially expressed genes in MIA- vs. MIA + DAE-treated groups. NC, rats given saline (vehicle); MIA, rats injected with MIA; MIA + DAE, rats injected with MIA and given DAE.

To understand how DAE improves OA phenotypes, we performed genome-wide analysis of gene expression in saline-, MIA-, and MIA + DAE-treated rats. First, we identified 3,008 DEGs in articular cartilage tissue from saline- and MIA-treated rats using as cutoff criteria a log2 fold change of >2 and an adjusted *p*-value of <0.05. Also, we identified 1,260 DEGs (872 upregulated and 388 downregulated) between MIA + DAE-treated rats and MIA-treated rats (Figure 4A). As shown in the volcanic plot depicting the relationship between fold change in gene expression and statistical significance (*p* < 0.05), DAE treatment modulated the expression of many genes (Figure 4B). Among these DEGs, the top 10 upregulated and top 10 downregulated genes are presented in Table 2. Collagen type I alpha 2 chain (COL1a2) was the gene with the highest fold (18.56-fold) increase in expression in the MIA group. Crispld1 (cysteine-rich secretory protein LCCL domain containing 1) was the most downregulated gene in MIA-treated rats. The top 10 genes with the highest fold increase in expression and the top 10 genes with the highest fold decrease in expression (ranked by fold change) after DAE treatment are shown in Table 3. MPO (myeloperoxidase) and DST (dystonin) were the top upregulated and downregulated genes, respectively. Among the DEGs selected according to our criteria, MPO was the gene most affected by DAE treatment. MPO activity is considered an effective diagnostic marker of oxidative damage and inflammation in OA and other immune diseases (Khan et al., 2018). Our results indicate that these genes were most affected by DAE treatment, but the role of these genes in OA requires further investigation.

**TABLE 2:** Top 10 upregulated and top 10 downregulated genes in the monosodium iodoacetate- (MIA-) treated rats vs. the saline-treated rats.

Gene ID	Gene name	Fold change	*p*-value
Upregulated			
Col1a2	Collagen type I alpha 2 chain	18.56	8.37E−04
Emp1	Epithelial membrane protein 1	14.15	1.91E−06
Fbln2	Fibulin 2	13.99	3.95E−24
Herc1	HECT and RLD domain containing E3 ubiquitin protein ligase family member 1	13.46	2.07E−09
Gpr116	Adhesion G protein-coupled receptor F5	13.39	1.77E−30
Erc1	ELKS/RAB6-interacting/CAST family member 1	13.20	2.76E−10
Gapdh	Glyceraldehyde-3-phosphate dehydrogenase	13.12	0.004432
Pld2	Phospholipase D2	13.11	2.28E−06
Impdh1	Inosine monophosphate dehydrogenase 1	13.08	9.75E−41
Il17ra	Interleukin 17 receptor A	12.90	3.53E−02
Downregulated			
Crispld1	Cysteine-rich secretory protein LCCL domain containing 1	−14.52	6.47E−20
Mpo	Myeloperoxidase	−13.88	7.58E−03
Mia	MIA SH3 domain containing	−13.78	4.31E−07
Cilp	Cartilage intermediate layer protein	−13.77	2.31E−02
Plxnb1	Plexin B1	−13.53	2.33E−02
Ddr2	Discoidin domain receptor tyrosine kinase 2	−13.30	3.51E−04
Mug1	Murinoglobulin 1	−12.84	4.47E−05
Ncoa6	Nuclear receptor coactivator 6	−12.83	2.95E−07
Hhipl2	HHIP like 2	−12.76	1.77E−15
Leprel1	Prolyl 3-hydroxylase 2	−12.74	1.76E−04

**TABLE 3:** Top 10 upregulated and top 10 downregulated genes in *Dipsacus asperoides* ethanolic extract- (DAE-) treated rats.

Gene ID	Gene name	Fold change	*p*-value
Upregulated			
Mpo	Myeloperoxidase	14.02	7.26E−03
Ank1	Ankyrin 1	13.51	1.85E−03
Ddr2	Discoidin domain receptor tyrosine kinase 2	12.73	4.88E−04
Pald1	Phosphatase domain containing, paladin 1	12.52	1.74E−03
Scap	SREBF chaperone	12.52	4.50E−03
Wdfy4	WDFY family member 4	12.51	7.84E−03
Fndc1	Fibronectin type III domain containing 1	12.44	1.75E−03
Braf	B-Raf protooncogene, serine/threonine kinase	12.32	4.00E−05
Ptprk	Protein tyrosine phosphatase, receptor type, K	12.26	4.29E−08
Dmxl1	Dmx-like 1	12.24	6.27E−03
Downregulated			
Dst	Dystonin	−14.32	8.15E−03
Dock10	Dedicator of cytokinesis 10	−12.34	5.68E−04
Htt	Huntingtin	−12.33	2.20E−02
Agrn	Agrin	−11.96	1.05E−03
Tp53inp2	Tumor protein p53 inducible nuclear protein 2	−11.63	5.81E−03
Anpep	Alanyl aminopeptidase, membrane	−11.48	1.26E−06
Ankrd50	Ankyrin repeat domain 50	−11.39	8.04E−03
Stag2	Stromal antigen 2	−11.27	1.35E−06
LOC691387	Similar to HBxAg transactivated protein 2	−11.23	2.34E−02
Gatad2a	GATA zinc finger domain containing 2A	−11.21	2.34E−02

Next, we explored the functions and signaling pathways of the genes whose expression was differentially affected by DAE treatment using Ingenuity Pathway Analysis. Based on a cutoff *p*-value of 0.05, 200 canonical pathways were associated with DEGs between saline and MIA groups, with the most significant pathways being OA pathways (Chun et al., 2021). The most significant canonical pathways associated with DEGs between MIA- and MIA + DAE-treated groups were GP6 and WNT/β-catenin signaling (Table 4). This result suggests that these specific pathways are important in the molecular mechanism by which DAE treatment reduces OA in rats.

**TABLE 4:** The 10 most significant canonical pathways affected by *Dipsacus asperoides* ethanolic extract (DAE) treatment.

Ingenuity Canonical Pathways	(*p*-value)	Ratio	*z*-score	Molecules
GP6 signaling pathway	4.84	0.150	3.15	ATM,COL11A2,COL13A1,COL1A2,COL23A1,COL28A1,COL2A1, COL9A1,COL9A2,GP6,GRAP2,ITGA2B,LAMA2,LAMC3,LYN, PIK3CB,PIK3CG,RASGRP2
Wnt/β-catenin signaling	3.23	0.110	−1.21	BTRC,CDH1,CREBBP,CSNK1D,FRZB,GNAO1,LEF1, MAP4K1, MARK2,PPP2R5E,SFRP2,SFRP5,SOX5,SOX6, TLE4, TP53, WIF1, WNT16,WNT9B
Salvage pathways of pyrimidine ribonucleotides	3.17	0.134	2.50	BRAF,CMPK2,CSNK1D,DAPK1,DMPK,GRK6,HIPK1,NME6,PAK3, PCK1,PRKAA2,SGK1,UPRT
Tec kinase Signaling	3.05	0.109	2.67	ACTA1,ATM,BMX,FGR,GNAO1,GNAS,GNAZ,GNG12, GNG2,YN, MS4A2, PAK3, PIK3CB, PIK3CG, PTK2B, RHOH,STAT2,VAV2
PI3K signaling in B lymphocytes	2.62	0.109	1.16	C3,CAMK2G,CD19,CD40,CD79A,CD79B,CR2,FCGR2B, LYN, NOTUM,PIK3CB,PIK3CG,PLEKHA4,PTPRC,VAV2
Pyrimidine ribonucleotides interconversion	2.47	0.167	2.65	CANT1,CMPK2,HNRNPA1,NME6,NUDT5,PCK1,SMARCAL1
Pyridoxal 5′-phosphate salvage pathway	2.46	0.138	1.67	BRAF,CSNK1D,DAPK1,DMPK,GRK6,HIPK1,PAK3,PRKAA2,SGK1
Pyrimidine ribonucleotides de novo biosynthesis	2.36	0.159	2.65	CANT1,CMPK2,HNRNPA1,NME6,NUDT5,PCK1,SMARCAL1
ILK signaling	2.33	0.094	2.00	ACTA1,ATM,CASP3,CDH1,CFL2,CREBBP,FLNC,LEF1,LIMS1, LIMS2,MYH1,MYH14,MYH4,PARVB,PIK3CB,PIK3CG,PPP2R5E, RHOH
RhoGDI signaling	2.26	0.094	−0.83	ACTA1,ARHGAP8/PRR5-ARHGAP8, ARHGEF10, ARHGEF11, ARHGEF7,CDH1,CFL2,CREBBP,ESR1,GNAO1,GNAS,GNAZ,GNG12,GNG2,PAK3,PIP5K1C,RHOH

### Validation of RNA Expression by Real-Time qPCR

**FIGURE 5:**
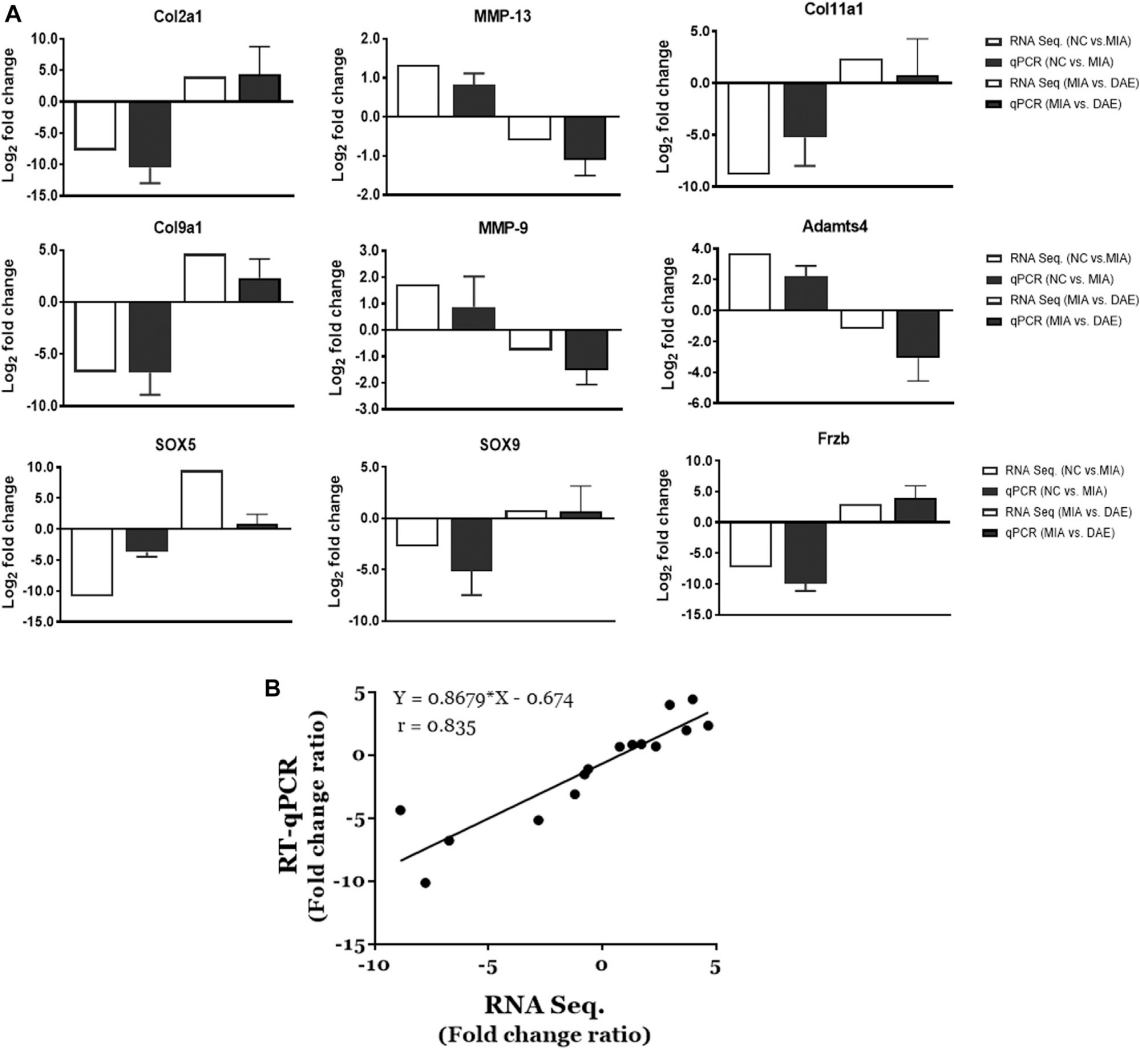
Validation of the mRNA expression of selected genes induced by monosodium iodoacetate (MIA) and *Dipsacus asperoides* ethanolic extract (DAE) in articular cartilage tissue. **(A)** Comparison of log2 fold changes in the expression of selected genes measured by RNA-seq and RT-qPCR in saline- vs. MIA-treated rats and MIA- vs. MIA + DAE-treated rats. RNA-seq data are presented as log_2_-transformed mean fold changes in gene expression. The RT-qPCR data are shown as log_2_-transformed mean fold changes± SD (*n* = 3–5 per group). NC, rats given saline (vehicle); MIA, rats injected with MIA; MIA + DAE, rats injected with MIA and given DAE. **(B)** The correlation between gene expression data measured by RNA-seq and RT-qPCR. The Pearson correlation coefficients and linear regression line are indicated.

The RNA sequencing-based expression data were validated by real-time qPCR of randomly selected genes associated with OA-related canonical pathways. As shown in Figure 5A, gene expression patterns determined by RNA-seq and qPCR analysis were comparable. The relative fold changes in mRNA expression of MMP9, MMP13, and Adamts4 were higher in the MIA-treated group than in the saline-treated group and lower in MIA + DAE-treated group than in the MIA-treated group. Additionally, some genes that were downregulated in the saline-treated compared with the MIA-treated group (Col2A1, Col9A1, Col11A1, SOX5, SOX9, and Frzb) were upregulated in the MIA-treated group compared with MIA + DAE-treated group. Comparisons of selected gene expression changes in saline-treated and MIA-treated rats, and MIA-treated and MIA/DAE-treated rats by RT-qPCR, were consistent with RNA-seq data and adjusted in the same direction. The correlation coefficient (*R* value) between mRNA expression values obtained by RNA sequencing and those obtained by RT-PCR was 0.835, indicating a high degree of concordance between the two methods (Figure 5B).

### Effect of *Dipsacus asperoides* Ethanolic Extract on MMP9 Expression in Knee Joint Tissue in Osteoarthritis Rats

**FIGURE 6:**
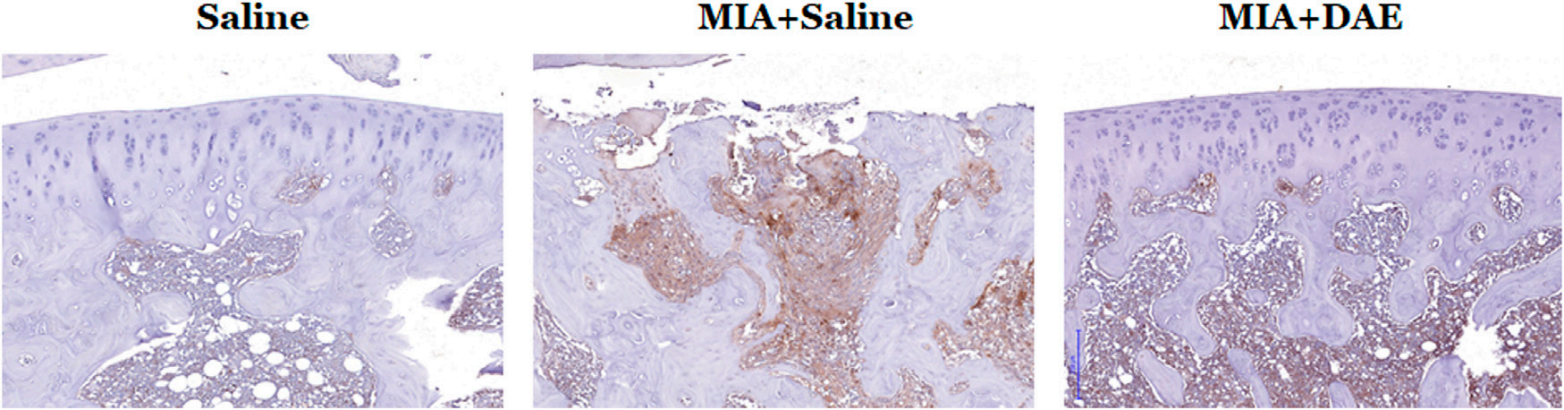
Effects of *Dipsacus asperoides* ethanolic extract (DAE) on the expression of MMP9 in knee joint lesions of MIA-induced OA rats. Representative images of immunohistochemical staining of MMP9 expression in knee joint tissue. Scale bars = 200 μm. Saline, rats given saline (vehicle); MIA + Saline, rats injected with MIA and given saline; MIA + DAE, rats injected with MIA and given DAE.

We explored important OA-related genes whose expression was affected by DAE treatment at the protein level by performing immunohistochemistry on arthritic knee joint tissues. Expression of MMP9, an important catabolic enzyme in OA progression, was higher in MIA-treated rats than in saline-treated rats but was lower in DAE-treated rats than MIA-treated rats. These changes in expression were observed at both the RNA and protein levels in articular cartilage tissues (Figure 6), suggesting that DAE treatment partially protected rats against OA by reducing MMP9 levels.

## Discussion

*Dipsacus asperoides,* a traditional herbal medicine, has long been used in China and Korea for the treatment of bone diseases such as bone fracture, osteoporosis, and rheumatoid arthritis (Wong et al., 2007; Gong et al., 2019). According to recent studies, *Dipsacus asperoides* has beneficial therapeutic effects on inflammation, osteoporosis, and joint disease (Liu et al., 2016a; Yue et al., 2017; He et al., 2019; Siu et al., 2019). However, the effects of *Dipsacus asperoides* on OA have not yet been reported. Therefore, this present study investigated the effects of DAE in MIA-induced OA in rats using RNA-seq analysis. Our results showed that DAE protected joints from the effects of OA in this model. In MIA-induced OA, MIA disrupts chondrocyte glycolysis by inhibiting glygeraldehyde-3-phosphate dehydrogenase activity, resulting in disruption of chondrocyte metabolism and initiation of cartilage degeneration (Pitcher et al., 2016). The MIA-induced animal model used in our study is one of the most commonly used chemically induced animal models of OA and has the advantages of rapid onset and minimal invasiveness, with characteristics similar to those of human OA (Combe et al., 2004; Kim et al., 2018). In rats treated with DAE, the knee joint diameter was effectively reduced, and overall improvements in the structural and histological features of the knee joint (based on morphological images and histopathological results) were observed. In our previous study, the effects of DAE on OA were evaluated by including indomethacin, a nonsteroidal anti-inflammatory drug, as a positive control. The results showed that DAE treatment was as effective as indomethacin (1 mg/kg) treatment in increasing the weight-bearing ability of hind paws and suppressing the serum levels of TNF-α and IL-1β (Chun et al., 2021a). Therefore, these results showed that DAE protects the joint against the effects of OA.

Omics techniques, including transcriptomics, can be used to guide the development of new therapeutic strategies and enable us to understand the effects and mechanisms of natural products (Guo et al., 2017; Seo et al., 2018). There have been many efforts to use gene expression profiling to identify therapeutic target genes related to OA and pharmacological mechanisms of herbal medicines. Transcriptomic analysis of the effects of natural products is becoming a popular approach for researching the treatment of diseases manifested by complex and multifaceted processes, such as OA (Thysen et al., 2015; Pandey et al., 2016). To understand the recovered OA phenotypes induced by DAE treatment, we used RNA-seq analysis and identified a transcriptome profile associated with DAE treatment in MIA-induced OA articular cartilage. This identified a total of 1,260 DEGs (872 upregulated and 388 downregulated) affected by DAE treatment in the MIA-induced OA model. Of these DEGs, Col1a2, a type I collagen that is the most common fibrillar collagen found in connective tissue, was the gene whose expression increased the most in the presence of MIA. The mRNA expression of Col1a2 is elevated in the bone of OA patients (Truong et al., 2006), and its function as a major gene involved in the OA process should be further investigated.

Functional analysis of DEGs affected by DAE showed that the canonical pathways most specifically linked to the DEGs were GP6 and WNT/β-catenin signaling. The Wnt/β-catenin signaling pathway directly affects bone, cartilage, and synovial tissue, regulating the development and progression of OA, and so therapeutically targeting this pathway could be a way to reduce these pathologies (Zhou et al., 2017). GP6 is a member of the immunoglobulin superfamily and is expressed in platelets and their precursor cells megakaryocytes. It acts as a major signal receptor for collagen, and activation of GP6 by collagen induces platelet activation and thrombus formation (Rivera et al., 2009); however, further studies are needed to determine the role of GP6 in OA. Our results showing that GP6 and WNT/β-catenin signaling are linked to DAE treatment suggest that these specific pathways represent important potential molecular mechanisms that can be regulated by DAE treatment. Therefore, our results suggest that DAE has a beneficial effect on OA by modulating various signaling pathways, including WNT/β-catenin signaling.

Our gene expression results showed that expression of gene encoding matrix-degrading enzymes (e.g., MMP9, MMP13, and Adamts4) were increased by MIA treatment and decreased by DAE treatment. By contrast, expression of cartilage collagen genes (e.g., Col2A1, Col9A1, and Col11A1) and SOX5, SOX9, and Frzb genes was decreased by MIA treatment and increased by DAE treatment. The results of RNA-seq analysis were confirmed by the results of qPCR analysis. In addition, we validated our results by comparing the RNA-seq data with the qPCR data for selected genes. The expression patterns of MMP9, an extracellular matrix-degrading enzyme involved in articular cartilage degeneration in OA (Meszaros and Malemud, 2012), were also validated at both the RNA and protein levels. Further studies are needed to determine the mechanisms involved in the protective effects of DAE.

Our phytochemical analysis of DAE components showed that DAE contained loganic acid, chlorogenic acid, loganin, sweroside, isochlorogenic acid A, and akebia saponin D. Phytochemical studies of *Dipsacus asperoides* have identified chemical compounds including triterpene saponins, iridoids, phenolics, and other compounds (Zhao and Shi, 2011). Several components of *Dipsacus asperoides* including saponins and iridoids may have biological effects (Kim et al., 2011; Zhang et al., 2019). One saponin, akebia saponin D, was identified as the main component of DAE in previous studies (Zhao et al., 2013; Shin et al., 2019); it has anti-inflammatory and antinociceptive actions in several cellular and animal models (Gong et al., 2019). Moreover, iridoids, which are present as secondary metabolites in various medicinal plants, are well known to exhibit anti-inflammatory effects (Viljoen et al., 2012). Loganin, an iridoid glycoside, counteracts OA progression by attenuating IL-1β-induced apoptosis and extracellular matrix catabolism in rat chondrocytes via regulation of PI3K/Akt signaling (Yang et al., 2019). In addition, sweroside protects against OA in rat cartilage by inhibiting NF-κB and mTORC1 signaling (Zhang et al., 2019). Chlorogenic acid has antiarthritis effects in an OA model by alleviating cartilage degradation through regulation of matrix-degrading enzymes (Chen et al., 2011). Although DAE is mainly composed of saponins and iridoides, which have various pharmacological activities, research into the OA-active compounds of DAE is still insufficient, and further studies are needed to reveal the effects of these compounds on OA.

## Conclusion

Our results showed that DAE treatment ameliorates OA disease phenotypes; DAE treatment reduced joint diameter and prevented changes in the structural and histological features associated with OA, thereby confirming that it protected against OA. Based on the results of gene expression profiles and pathway analysis, we found that DAE-induced gene expression changes are linked to several canonical signaling pathways, including WNT/β-catenin signaling. Taken together, the present results suggest a molecular mechanism, involving gene expression changes, by which DAE protects against MIA-induced OA in rats.

## Data Availability

The datasets presented in this study can be found in online repositories. The names of the repository/repositories and accession number(s) can be found below: SRA, PRJNA673059

## Footnotes

1 Chun, J. M., Lee, A. Y., Moon, B. C., Choi, G., and Kim, J. S. (2021). Evaluation of the effects of Dipsacus asperoides extract and Phlomis umbrosa extract on osteoarthritis in monosodium iodoacetate-induced rats. In preparation

## Abbreviations

OA, Osteoarthritis; MIA, Monosodium iodoacetate; DAE, *Dipsacus asperoides* ethanolic extract; SCAR, Sequence characterized amplified region; LOD, Limit of detection; LOQ, Limit of quantification; H&E, Hematoxylin and eosin; OARSI, Osteoarthritis Research Society International; RNA-seq, RNA sequencing; DEGs, Differentially expressed genes; qPCR, Quantitative polymerases chain reaction; ANOVA, One-way analysis of variance; COL1a2, Collagen type I alpha 2 chain.
