# Supplementary figures and images for: Effects of Dipsacus asperoides Extract on Monosodium Iodoacetate–Induced Osteoarthritis in Rats Based on Gene Expression Profiling
Source: Front Pharmacol. 2021 Apr 13;12:615157. doi: 10.3389/fphar.2021.615157 (PMC8076797; doi:10.3389/fphar.2021.615157)

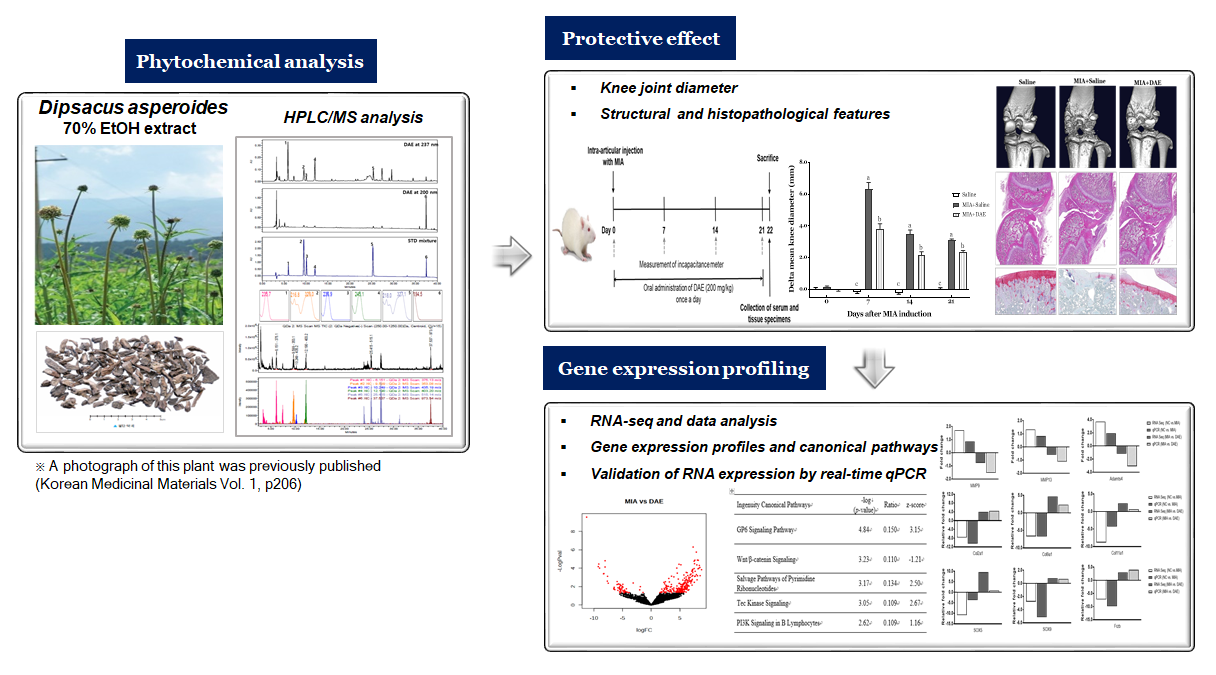

Supplement: Supplementary file 1 [file image1.tif]
