# Supplementary material for: Effects of Dipsacus asperoides Extract on Monosodium Iodoacetate–Induced Osteoarthritis in Rats Based on Gene Expression Profiling
Source: Front Pharmacol. 2021 Apr 13;12:615157. doi: 10.3389/fphar.2021.615157 (PMC8076797; doi:10.3389/fphar.2021.615157)
Supplement: Supplementary file 2 [file table1.docx]

Supplementary data

| **Genes** |  | **Primer sequence** | **Accession number** | **Product length** |
| --- | --- | --- | --- | --- |
| MMP-9 | Forward  Reverse | GATCCCCAGAGCGTTACTCG  GTTGTGGAAACTCACACGCC | NM_031055.1 | 132 |
| MMP-13 | Forward  Reverse | ACCCAGCCCTATCCCTTGAT  TCTCGGGATGGATGCTCGTA | NM_133530.1 | 178 |
| ADAMTs4 | Forward  Reverse | ACCGATTACCAGCCTTTGGG  CCGACTCCGGATCTCCATTG | NM_023959.1 | 168 |
| COL2A1 | Forward  Reverse | GGCCAGGATGCCCGAAAATTA  ACCCCTCTCTCCCTTGTCAC | NM_012929.1 | 153 |
| COL9A1 | Forward  Reverse | TTCCGGATGACTGGAAGCAC  GTTTGGAGACTCCCGTCCAG | NM_001100842.1 | 149 |
| COL11A1 | Forward  Reverse | GTCTTGTCTACCTGGATTGGTCA  TGAAGGTTAACATGCAAGGGT | NM_013117.1 | 132 |
| SOX5 | Forward  Reverse | CAAGTACAAACCCAGGCCGA  ACAAGGGGAGGATCGTCTGT | NM_001271267.1 | 188 |
| SOX9 | Forward  Reverse | GCAAACACGTTGCAAATGGC  AAGTCCAAACAGGCAGGGAG | NM_080403.1 | 128 |
| Frzb | Forward  Reverse | TGAAGAGCGCTCCAGGTTAC  GGATTAGCGTTCCTGCCAGA | NM_001100527.1 | 171 |
| β -actin | Forward  Reverse | ACTCTGTGTGGATTGGTGGC  CGCAGCTCAGTAACAGTCCG | NM_031144.3 | 140 |

SUPPLEMENTARY TABLE 1. Sequences of real-time PCR primer
